# Up-Regulation of 91H Promotes Tumor Metastasis and Predicts Poor Prognosis for Patients with Colorectal Cancer

**DOI:** 10.1371/journal.pone.0103022

**Published:** 2014-07-24

**Authors:** Qiwen Deng, Bangshun He, Tianyi Gao, Yuqin Pan, Huiling Sun, Yeqiong Xu, Rui Li, Houqun Ying, Feng Wang, Xian Liu, Jie Chen, Shukui Wang

**Affiliations:** 1 Central Laboratory, Nanjing First Hospital, Nanjing Medical University, Nanjing, Jiangsu, China; 2 Department of Life Sciences, Nanjing Normal University, Nanjing, Jiangsu, China; 3 Medical college, Southeast University, Nanjing, Jiangsu, China; University of Kentucky College of Medicine, United States of America

## Abstract

**Background:**

Long noncoding RNAs (lncRNAs) play widespread roles in gene regulation and cellular processes. However, the functional roles of lncRNAs in colorectal cancer (CRC) are not yet well elucidated. The aim of the present study was to measure the levels of lncRNA 91H expression in CRC and evaluate its clinical significance and biological roles in the development and progression of CRC.

**Methods:**

91H expression and copy number variation (CNV) were measured in 72 CRC tumor tissues and adjacent normal tissues by real-time PCR. The biological roles of 91H were evaluated by MTT, scratch wound assay, migration and invasion assays, and flow cytometry.

**Results:**

91H was significantly overexpressed in cancerous tissue and CRC cell lines compared with adjacent normal tissue and a normal human intestinal epithelial cell line. Moreover, 91H overexpression was closely associated with distant metastasis and poor prognosis in patients with CRC, except for CNV of 91H. Multivariate analysis indicated that 91H expression was an independent prognostic indicator, as well as distant metastasis. Our in vitro data indicated that knockdown of 91H inhibited the proliferation, migration, and invasiveness of CRC cells.

**Conclusions:**

91H played an important role in the molecular etiology of CRC and might be regarded as a novel prognosis indicator in patients with CRC.

## Introduction

Colorectal cancer (CRC) is a common cause of cancer death in the world due to late tumor presentation and rapid progression, with about 14.1 million new cancer cases and 8.2 million cancer deaths in 2012 worldwide [1]. In economically developing countries, CRC is becoming more prevalent, especially in China [2]. Despite substantial progress achieved in diagnosis and treatment for CRC in recent years, the overall 5-year survival rate remains unsatisfactory due to metastasis leading to poor outcomes [3]. Therefore, to seek novel molecular markers or factors is necessary and urgent for the early detection before distant metastasis appears and predict prognosis in patients with CRC.

Recent studies have revealed that lncRNAs, >200 nucleotides in length, play a critical role in cancer development and progression. Despite less well understanding of lncRNAs compared with microRNAs [3], [4], [5], accumulating evidences indicate that lncRNAs-mediated biology could be involved in regulation of diverse cellular processes, such as cell growth and apoptosis, stem cell pluripotency and development, meiotic entry and telomere length [6], [7], [8], [9], [10], [11]. However, similar to protein-coding genes and micorRNAs, lncRNAs may function as oncogenes or tumor suppression, and aberrant expression of them was associated with carcinogenesis. High expression of PVT-1 expression in cancerous tissues was regarded as an independent risk factor for overall survival of CRC patients [12], and HOTAIR, functioning as an oncogene or a tumor suppressor, was implicated in epigenetic regulation for cancers [7], [13], which was considered as a strong prognosis maker that contributed to predict metastasis and patients’ survival in primary breast cancer [7].

91H, H19 antisense RNA, was a novel lncRNA that was located on the position of the H19/IGF2 locus (Accession number NC_000011.9) with 119.329 kbs in length. The transcript of 91H was overexpressed in human breast cancer which was involved in the regulation of IGF2 expression in trans [14]. However, few studies have investigated the functions of 91H in other forms of human cancer, including CRC. To explore the potential biological functions of 91H in the development and progression of CRC, the current study was carried out by examining the expression pattern of 91H in CRC tumor tissues and adjacent normal tissues and investigating the biological functions by assessment of the invasiveness, migration, and apoptosis of CRC cells with 91H knockdown in vitro.

## Materials and Methods

### Clinical samples and cell lines

72 tumor tissues and matched adjacent normal tissues were obtained from enrolled CRC patients who underwent surgery at Nanjing First Hospital Affiliated to Nanjing Medical University, between 2011 and 2014. In particular, adjacent normal tissues were taken 5–10 cm away from the tumor tissues. All specimens were immediately frozen in liquid nitrogen after surgery and stored at −80°C until DNA and RNA extraction. No patients received chemotherapy or radiotherapy at pre-operation. Clinical information of these patients was collected including age, sex, tumor location, TNM stage, tumor grade and microstellite instability (MSI) status as previous study [15] reported. The follow-up periods varied from 2 months to 3 years, with a mean of 26.6 months. All the samples were obtained with the patients’ written informed consent and were histologically confirmed. The medical ethics committee of the Nanjing First Hospital Affiliated to Nanjing Medical University approved the study.

Cell lines HCT8, HT29, HCT116, SW620 (human colon cancer cell lines) and FHC (normal human intestinal epithelial cell line) were obtained from Shanghai Cell Collection, Chinese Academy of Sciences. All above cell lines were maintained in DMEM (Hyclone, USA) containing 10% fetal bovine serum (FBS; Hyclone) and cultured at 37°C in a humidified atmosphere with 5% CO_2_.

### Extraction of total RNA and genomic DNA, cDNA synthesis, and qRT-PCR

Total RNA and genomic DNA (gDNA) were extracted from tissues and cell lines using the Trizol reagent (Invitrogen, CA) and E.Z.N.A. Tissue DNA Kit (Omega, USA) following the manufacturers’ protocol separately. cDNA was synthesized using the PrimeScript RT reagent Kit with gDNA Eraser (Takara, China) and was used for 91H expression level analysis by quantitative real-time PCR (qRT-PCR) with the following primers: forward, 5′- GCTTGTCAGTAGAGTGCGCC-3; and reverse, 5-CATCCAGTTGACCGAGCTTG-3. β-actin was used as an internal control with the following sequences: forward, 5-CAAGATCATTGCTCCTCCTGA-3; reverse: 5-AGTCCGCCTAGAAGCATTTG-3. The 2^−ΔΔCt^ method was performed to calculate the relative amount of 91H compared with β-actin expression. qRT-PCR was performed using the SYBR Premix Ex TagTM II (Takara, China) and ABI 7500 System (Applied Biosystems, USA).

### Copy number variation identification of 91H

Analysis of CNV for gDNA was also performed by qRT-PCR that described above method. RNase P was used as a reference gene. The copy number of 91H was counted following the equation 2×2^−ΔΔCT^.

### Transfection of siRNA

RNA interference was performed by using synthetic siRNA duplexes, as described by previous studies [16], [17]. Two synthetic siRNA duplexes (si-91H: si91H1, 5′-GGCGUCAUUCUGAUGGGACTT-3′ and si91H2, 5′- UUCAGGAGCUUAAGAUGCUTT-3′) corresponding to the 91H RNA sequences and a negative control (si-NC, 5′-CGUGGGUGGAUGCAUGGAUTT-3′) were transfected into HCT-116 cells with 400 pmol respectively using the Lipofectamine 2000 transfection reagent (Invitrogen, CA) in the 6-well plate. After 48 hours, 91H expression levels were examined via qRT-PCR.

### Cell proliferation assay

After transfection for 48 hours as described above, infected HCT-116 cells were subsequently harvested for cell proliferation assay (MTT assay) following the manufacturer’s protocol. Infected cells (1×10^4^) were plated in flat bottom 96-well plates supplemented with 100 µl DMEM per well. After incubation for 6, 24, 48, 72 and 96 hours respectively, 10 µl of MTT was added to each well, then the medium was removed after 4 hours of culture and subsequently supplemented with 150 µl DMSO per well. Colorimetric analysis was performed on a microplate reader at 490 nm wavelength. Each subgroup was repeated for five wells.

### Migration and Invasion assays

For migration assay, infected HCT-116 cells (1×10^5^ in 200 µl of serum-free DMEM), just as described previously, were seeded into the upper chamber of transwell plates in a 24-well format with 8 mm diameters (Corning Costar, USA). 600 µl of DMEM containing 5% FBS were added to the bottom chamber as a chemoattractant. After 24 hours of culture, cells were fixed with methanol and were stained with crystal violet. Remaining cells were removed from the top of the permeable membrane using a cotton swab. Then cells that migrated through the upper chamber were counted in five random fields under a light microscope, and the average value of five fields was expressed.

For invasion assay, the top chambers were coated with basement membrane Matrigel (40 mg/ml, Becton-Dickinson, USA) at 37°C for 30 min. Infected cells (3×10^5^) with serum-free DMEM were added into the top chambers, the bottom chambers were filled with DMEM containing 10% FBS. After 24 hours of incubation, the cells that invaded the reverse side of top chambers were fixed, stained, and calculated using a light microscope. Each invasion assay was performed in three or more replicates.

### Scratch wound assay

Six-well plates were coated with infected HCT-116 cells. Wounds were created in confluent cells using a 10 µl pipette tip at 48 hours post-transfection. Subsequently free-floating cells and debris were removed using PBS, and FBS-free medium was added. Then we observed the wound healing at different time periods and photographed representative scrape lines using the light microscope. Each experiment was repeated in triplicate.

### Apoptosis analysis

Cell apoptosis was determined using flow cytometry after staining with Annexin V-FITC and propidium iodide (PI, BD Bioscience, CA). HCT-116 cells were infected with si-91H or si-NC in 6-well plate. Apoptosis was analyzed after 48 hours infection. All apoptosis assays were performed in triplicate.

### Statistical analysis

Optimal cut-off levels of 91H in cancerous/noncancerous were calculated by applying the receiver operating characteristic curve analysis (ROC) [18], [19]. Comparison of continuous data was analyzed using an independent *t*-test, and categorical data were analyzed by chi-square test. Meanwhile, survival rates were calculated by Kaplan-Meier survival analysis and Log-rank test. A Cox proportional hazards model univariate and multivariate analysis were conducted to determine the impact of 91H expression and clinicopathologic parameters on overall survival (OS). Nomogram for OS was established by applying R software. Harrell’s concordance index (c-index) was used to estimate the predictive accuracy of it. Statistics with *P*-value<0.05 were considered as statistically significant. All these statistical calculations were performed using IBM SPSS 20.0 software (IBM, USA), R 3.0.3 software (Institute for Statistics and Mathematics, Vienna, Austria), OriginLab 8.5.1 software (OriginLab Corp, Northampton, USA) and GraphPad Prism 5 (GraphPad Software, USA).

## Results

### Correlation between 91H relative expression and clinicopathologic factors in CRC

**Figure 1:**
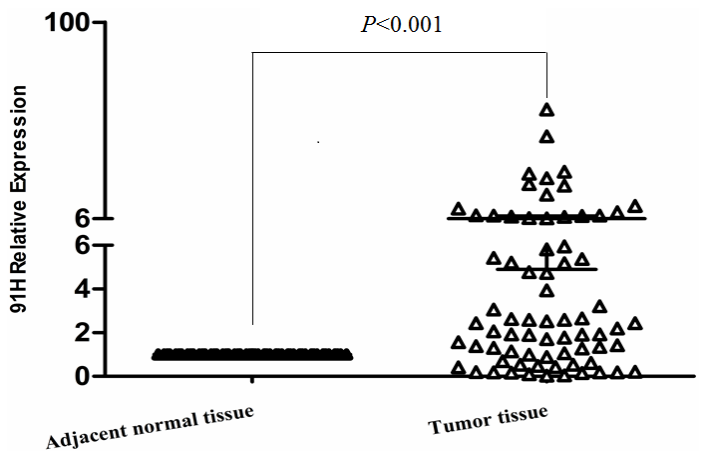
Comparison of 91H expression between tumor tissues and adjacent normal tissues from 72 patients with CRC by qRT-PCR.

**Figure 2:**
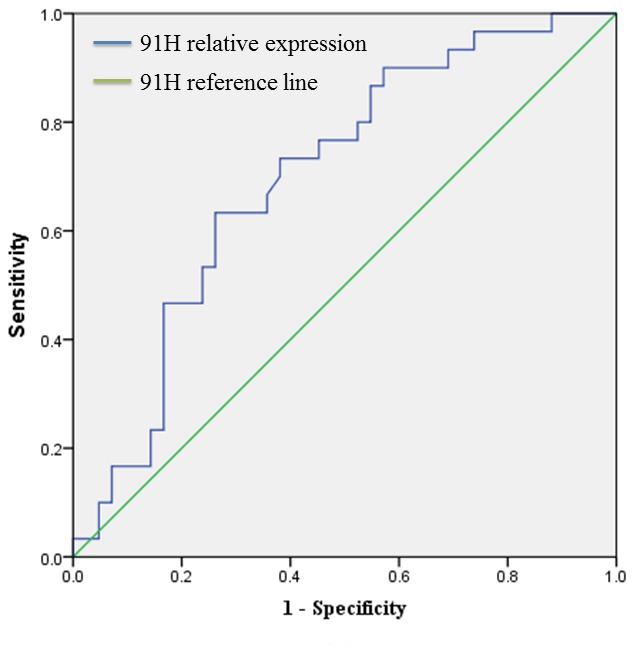
The optimal cut-off levels of 91H relative expression were determined based on the largest of sensitivity and specificity by receiver operating characteristic curve (ROC) analysis for overall survival.

**Figure 3:**
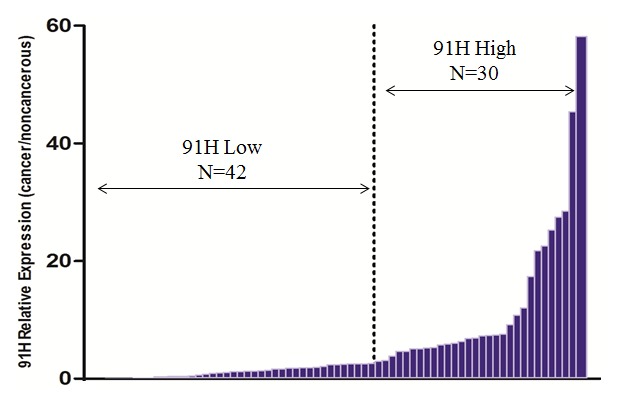
According to the optimal cut-off levels (2.86-fold), 72 patients with CRC were divided into two groups: ≥2.86 were considered as an elevated 91H expression group (n = 30); <2.86 were considered as a low 91H expression group.

**Figure 4:**
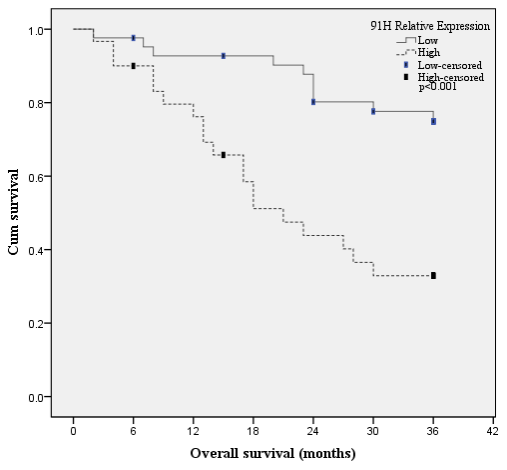
Kaplan-Meier overall survival curves based on 91H relative expression.

The 91H expression levels were determined by qRT-PCR for 72 cancerous and adjacent noncancerous tissues from CRC patients. 91H levels in cancerous tissues were obviously higher than those in the noncancerous tissues (*P*<0.001; Figure 1). ROC curve analysis revealed that the optimal cut-off levels for 91H expression were 2.86-fold for OS in cancerous/noncancerous (Figure 2). Then the 72 patients with CRC were divided into a high 91H expression group (n = 42) that 91H expression ratio ≥2.86 and a low 91H expression group (n = 30) that 91H expression ratio <2.86 (Figure 3), according to cut-off level. Clinicopathologic factors were compared between two 91H expression groups (Table 1). 91H expression was significantly correlated with distant metastasis (*P* = 0.027). However, 91H expression was hardly correlated with patients’ sex, age, tumor location, TNM stage, N stage or grade. To further estimate the correlation between 91H expression and prognosis of patients with CRC, Kaplan-Meier survival analysis and log-rank tests were performed using patients’ postoperative survival time. The results indicated that patients with high 91H expression had an obviously poorer prognosis than those with low 91H expression (*P*<0.001, Figure 4).

**Table 1:** Clinicopathological characteristics and 91H expression of CRC patients.

Factors	Tumor low expressionΔ(n = 42) N (%)	Tumor high expressionΔ(n = 30) N (%)	*P*
Sex			
Male	23 (54.8%)	19 (63.3%)	0.467^c^
Female	19 (45.2%)	11 (36.7%)	
Age (mean ±SD)	66.0±12.6	70.0±11.6	0.167^d^
<67 yrs	20 (47.6%)	11 (36.7%)	0.355^c^
≥67 yrs	22 (52.4%)	19 (63.3%)	
Tumor location			
Colon	28 (66.7%)	24 (80%)	0.213^c^
Rectum	14 (33.3%)	6 (20%)	
TNM^a^			
I–II	23 (54.8%)	12 (40%)	0.217^c^
III–IV	19 (45.2%)	18 (60%)	
T			
T1–T2	7 (16.7%)	1 (3.3%)	NA^e^
T3–T4	35 (83.3%)	29 (96.7%)	
N			
N0	24 (57.1%)	19 (63.3%)	0.597^c^
N1–N2	18 (42.9%)	11 (36.7%)	
M			
M0	35 (83.3%)	18 (60%)	**0.027** c
M1	7 (16.7%)	12 (40%)	
Grade^b^			
G1–G2	36 (85.7%)	23 (76.7%)	0.325^c^
G3	6 (14.3%)	7 (23.3%)	
MSI			
Stable	39 (92.9%)	25 (83.3%)	NA^e^
Unstable	3 (7.1%)	5 (16.7%)	

T, depth of tumor; N, lymph node; M, distant metastasis.

Δ 91H low and high expression groups were split using the cut-off value 2.86 by ROC curve.

a Tumor-node –metastasis (TNM) stage system according to AJCC classification [35].

b Grade 1 and 2 stand for high or middle differentiated tumor, grade 3 stands for poorly differentiated tumor.

c Two-sided chi-square test.

d Independent *t*-test.

e *P*-value not showed due to low number of CRC patients with T1–T2 and MSI unstable.

**Figure 5:**
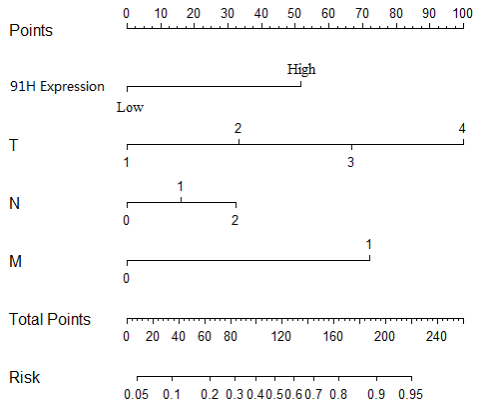
Postoperative nomogram with 91H expression and significant clinicopathologic parameters predicted the probability of CRC for 3-year death.

Unvariate analysis of overall analysis revealed that 91H relative expression, TNM stage, distant metastasis or grade were prognostic indicators (Table 2). Furthermore, 91H relative expression and distant metastasis were independent prognostic indicators for the overall survival rate of patients with CRC (HR: 3.66, *P* = 0.001; HR: 8.97, *P*<0.001) respectively by multivariate analysis (Table 2). In addition, prognostic nomogram with clinicopathologic factors and 91H expression was established to predict the probability that patients with CRC would die within 3 years of his initial surgery, assuming patients did not die of other causes first (Figure 5). Meanwhile, the nomogram’s predictive accuracy was measured via a concordance index (c-index). For CRC, nomogram containing 91H had more predictive accuracy than that without 91H (c-index: 0.90 versus 0.85 respectively).

**Table 2:** Cox proportional hazard analysis: impact of 91H expression and clinicopathological parameters on overall survival in CRC patients.

Risk factors	Category	Univariate analysis		Multivariate analysis	
		HR (95%CI)	*p* a	HR (95%CI)	*p* a
91H in tumor	Low (n = 42)/High (n = 30)	4.03 (1.86–8.73)	**<0.001**	3.66 (1.66–8.10)	**0.001**
Sex	M (n = 42)/F (n = 30)	0.76 (0.36–1.61)	0.475	1.35 (0.61–2.96)	0.457
Age (yrs)	<67 (n = 31)/≥67 (n = 41)	1.63 (0.76–3.51)	0.211	1.25 (0.52–3.04)	0.617
Tumor location	Colon (n = 52)/Rectum (n = 20)	0.61 (0.26–1.44)	0.260	0.96 (0.35–2.62)	0.938
TNM	I–II (n = 35)/III–IV (n = 37)	4.89 (1.99–12.06)	**0.001**	0.72 (0.15–3.54)	0.688
N	N0 (n = 43)/N1–N2 (n = 29)	1.78 (0.86–3.69)	0.122	1.86 (0.85–4.07)	0.122
M	M0 (n = 53)/M1 (n = 19)	9.44 (4.33–20.61)	**<0.001**	8.97 (4.05–19.88)	**<0.001**
Grade	G1–G2 (n = 59)/G3 (n = 13)	2.78 (1.29–6.00)	**0.009**	0.88 (0.34–2.28)	0.789

HR, hazard ratio; 95%CI, 95% confidence interval; N, lymph node; M, distant metastasis.

a Cox proportional hazards regression.

### DNA copy number variation (CNV) was not involved in up-regulation of 91H expression

**Figure 6:**
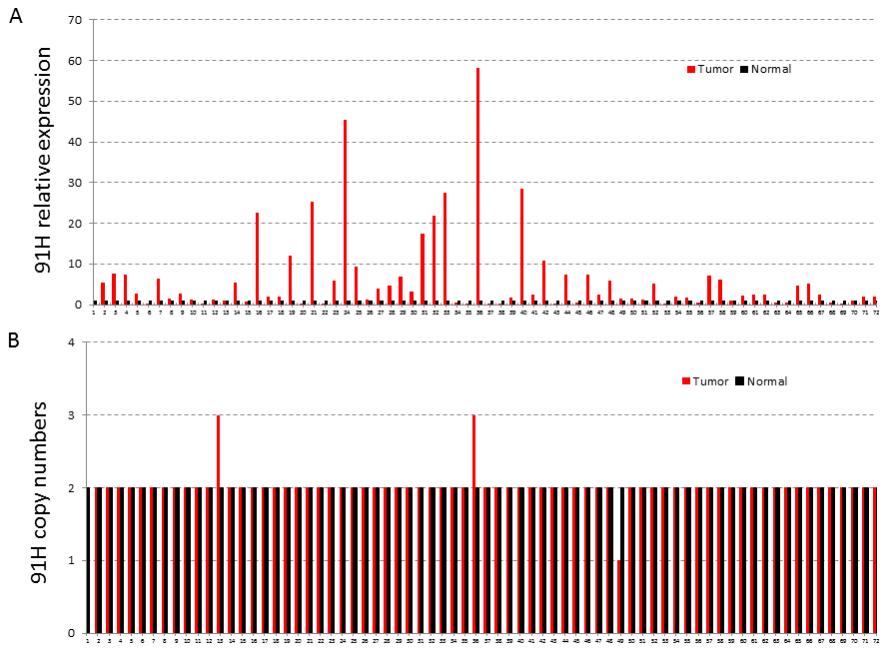
The relationship between copy number variation and 91H expression in tumor tissues and adjacent normal tissues. A, The 91H relative expression in matched cancerous and noncancerous tissues was measured by qRT-PCR. β-actin was considered as the internal control. B, The copy number variation of 91H in cancerous and noncancerous tissues was also measured by qRT-PCR. RNase P was used as a reference gene.

To determine whether 91H expression level in CRC was associated with CNV, the expression pattern of 91H was measured by qRT-PCR, and further estimated the concordance between 91H DNA copy number and 91H relative expression level. Only 2.8% (2/72) of paired normal and tumor tissues with CNV showed the overexpression of 91H. However, the aberrant expression of 91H was detected in 95.8% (69/72) of paired normal and tumor tissues without copy number alteration (Figure 6).

### 91H promoted proliferation, invasion and migration of CRC cells in vitro

**Figure 7:**
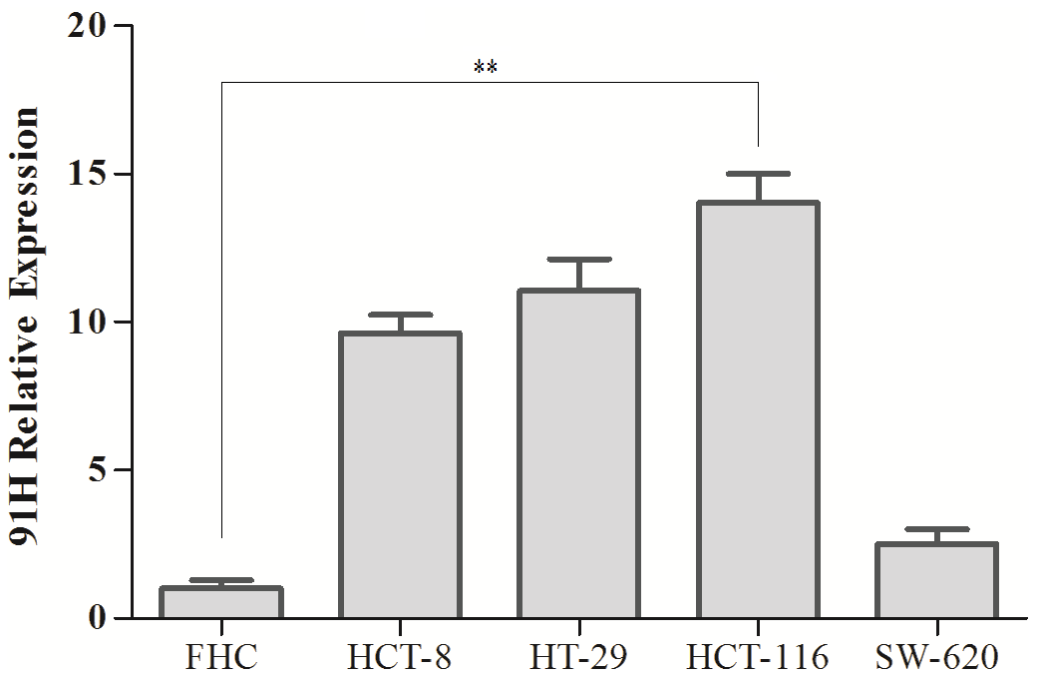
91H relative expression was quantified in four CRC cell lines and a normal human intestinal epithelial cell line by qRT-PCR (mean ± SEM; **P<0.01).

**Figure 8:**
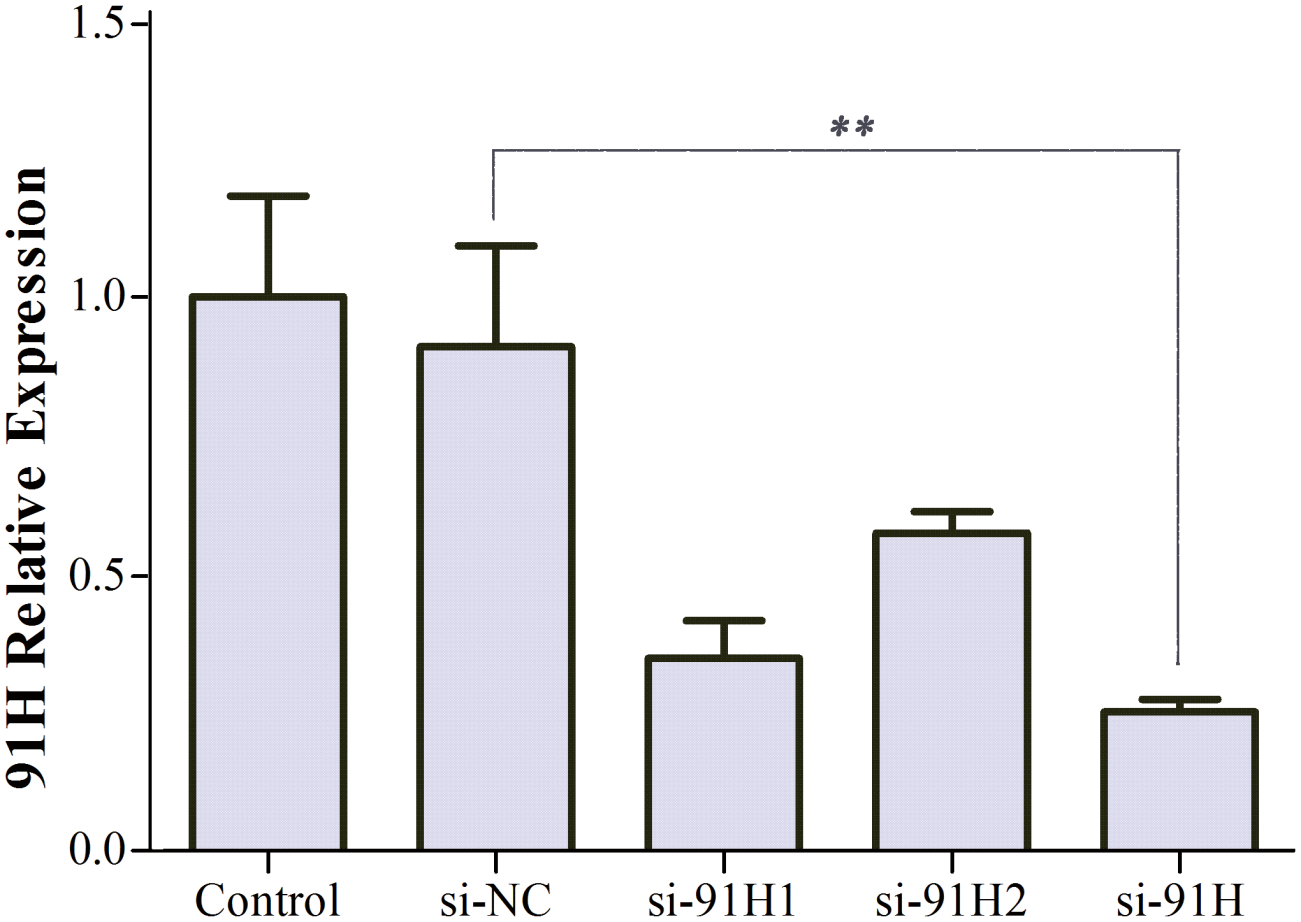
HCT-116 cells were transfected with si-NC and si-91H. After 48 h, 91H expression was effectively inhibited by qRT-PCR compared with si-NC (mean ± SEM; **P<0.01).

To evaluate the biological functions of 91H, 91H expression levels were measured in a variety of cell lines (FHC, HCT-8, HT-29, HCT-116, and SW-620) by qRT-PCR. As shown in Figure 7, 91H expression was detected the higher levels in HCT-8, HT-29 and HCT-116 compared with FHC, except for SW-620. Afterwards, HCT-116 cells were transfected with si-91H or si-NC. After transfection for 48 hours, 91H was effectively silenced in HCT-116 cells (Figure 8).

**Figure 9:**
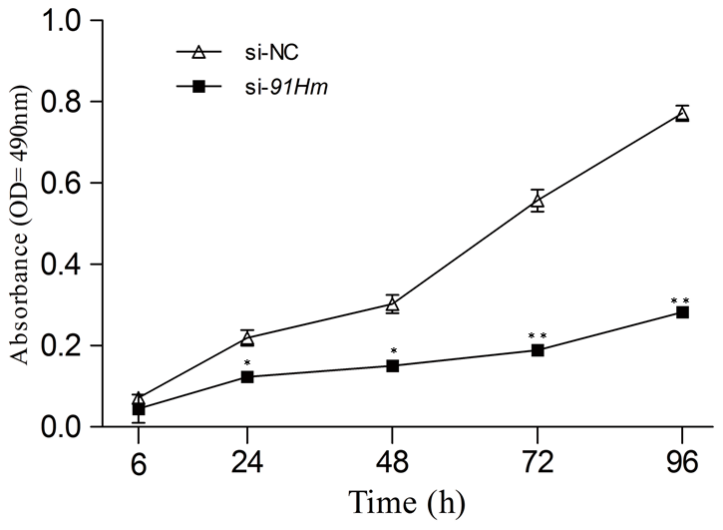
91H promoted the proliferation of HCT-116 cells via MTT assay (mean ± SEM; *P*<0.05).

**Figure 10:**
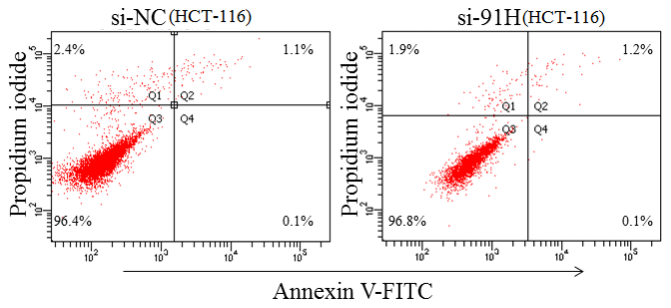
Apoptosis was investigated using cytometric analysis at 48-91H or si-NC. Data were representative of three separate experiments.

We next sought to determine whether 91H knockdown affected cell proliferation by MTT assay. The data, compared with HCT-116 cells infected with si-NC, showed that the proliferation of HCT-116 cells with si-91H was inhabited to 36.7% (*P*>0.05), 45.4% (*P*<0.05), 43.2% (*P*<0.05), 65.6% (*P*<0.01) and 65.3% (*P*<0.01) at 6, 24, 48, 72 and 96 h, respectively (Figure 9). In addition, the induction of apoptosis following 48 hours of treatment with si-91H or si-NC was measured using flow cytometry. HCT-116 cells with 91H knockdown did not obviously display apoptosis compared with those in control (Figure 10).

**Figure 11:**
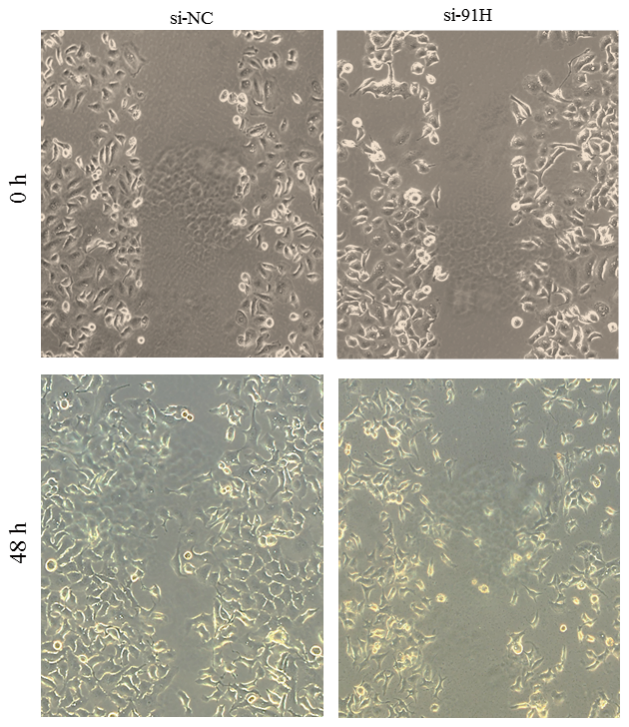
The scratch wound assay was assessed to cell motility. The knockdown of 91H inhibited HCT-116 cell motility.

**Figure 12:**
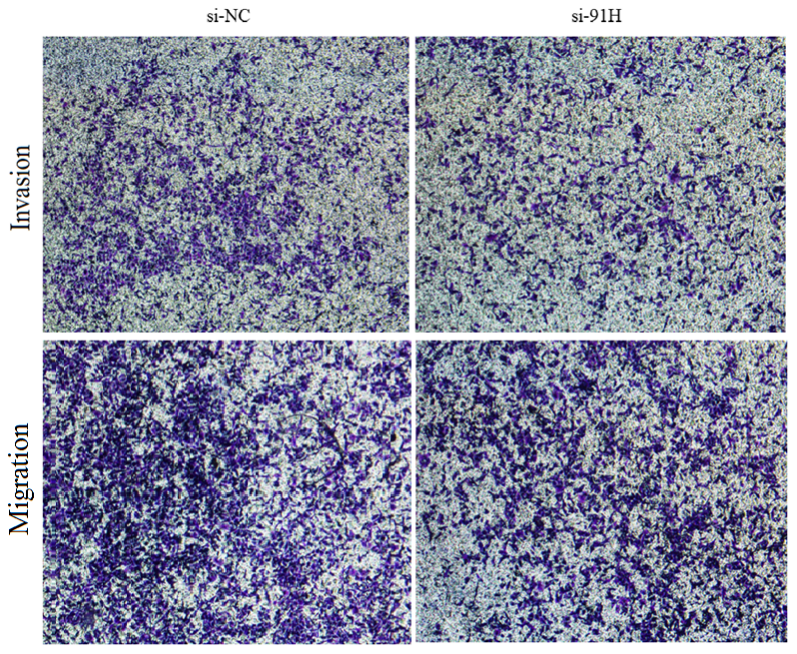
91H promoted invasion and migration of HCT-116 cells based on transwell assay.

**Figure 13:**
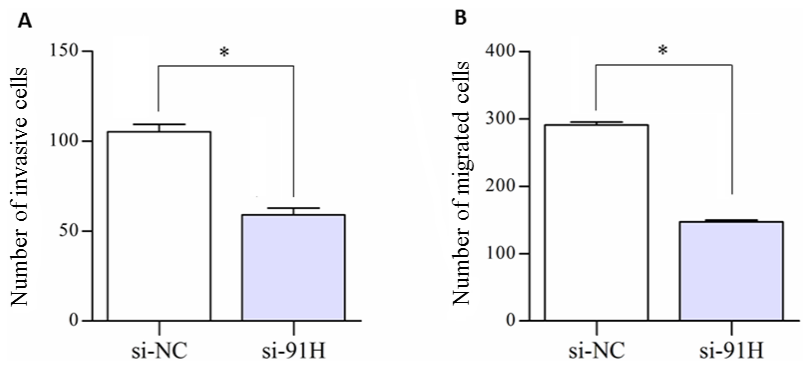
The number of cells that invaded or migrated through the chamber was evaluated in 5 fields for each experimental group and averaged. The number of invasive or migrated cells for each experimental group was counted as the average of 5 five fields of vision under a microscope (*P<0.05).

Subsequently, to access the effects of 91H suppression on HCT-116 cells motility, a scratch wound assay was performed to gauge the effect of 91H knockdown on cell motility. 91H suppression resulted in attenuating motility of HCT-116 cells. In particular, compared with infected cells with si-NC, the wound recovery was significantly delayed in siRNA-infected cells (Figure 11). Furthermore, migration and invasion assays were used to further access this effect of 91H knockdown on migration and invasiveness of CRC cells. The results revealed that the migration and invasiveness were significantly inhibited in infected cells with si-91H, compared with si-NC-infected cells (Figure 12). Moreover, HCT-116 cell migration and invasion were decreased by 49.4% (P<0.05) and 43.9% (P<0.05), respectively, following 91H suppression (Figure 13). These findings indicated that 91H inhibition might be closely correlated with proliferation, migration, and invasion of CRC cell lines.

## Discussion

As a novel molecular star for lncRNA, accumulating studies have focused on the impact of lncRNA on cancer pathogenesis, and could provide new insights into the biology of these cancers [20], [21], [22]. Despite substantial progress of lncRNAs in cancer pathogenesis, biological roles of lncRNAs are not clear in CRC. To explore the biology of lncRNA in CRC, this study was performed to firstly investigate the biological roles of 91H in CRC progression and assess the effect of 91H on clinicopathological characteristics and prognosis.

In this study, the data revealed that 91H expression levels in CRC tissue were significantly higher than those in corresponding noncancerous tissue. The result was identified in vitro with CRC cell lines and normal human intestinal epithelial cell line (Figure 7). Moreover, the overexpression of 91H was associated with distant metastasis of clinicopathologic factors (*P*<0.05). 91H was detected to be stable expression in breast cancer cell lines, and it was observed to be up-regulated in patients with primary breast cancer [14]. More important, in mouse myoblasts, 91H was determined to be implicated in co-regulation of genes at the H19/IGF2 locus contributing to carcinogenesis and cancer progression [16]. However, 91H expression was not associated with distant metastasis of clinicopathologic factors in esophageal squamous cell carcinoma (ESCC) [23]. The inconsistent conclusions to link 91H expression with clinicopathological factors may be due to different histopathologic types. In addition, using in vitro data, we showed that 91H knockdown inhibited the cell motility, migration and invasiveness of CRC cells. These results indicated that 91H might play a potential role in promoting metastasis of CRC.

Furthermore, we firstly described the evidence that high 91H expression levels in tumor tissue were related with poor prognosis, and 91H relative expression levels were intimately interrelated with poor clinical outcome of patients, indicating the expression of 91H could be served as a valuable independent prognostic biomarker independent on major clinicopathological characteristics, except for metastasis status. In fact, previous studies indicated that lncRNAs were also considered as molecular biomarkers in cancers. PVT-1, generating antiapoptotic activity in CRC, was identified as a novel prognostic indicator for CRC patients [12], [24]. Meanwhile, HOTAIR was involved in tumor progression and was regarded as a novel epigenetic molecular biomarker in patients with ESCC or CRC [18], [25], [26]. Therefore, we consider that 91H might be a novel potential prognostic indicator in patients with CRC.

Generally, nomograms have been developed in most of cancer types [27], [28], [29], which create a simple graphical representation of a statistical predictive model that generates a numerical probability of a clinical event [30]. Moreover, the ability of nomograms to produce individualized predictions enables their use in the identification and stratification of patients for participation in clinical trials [30]. In this study, based on 91H relative expression in tumor and clinicopathological factors, a predictive nomogram was performed to predict the probability that postoperative patients would die of CRC within 3 years, except for dying of another cause first. Meanwhile, nomogram containing 91H had more predictive accuracy than that without 91H (c-index: 0.90 versus 0.85 respectively), indicating that 91H expression in tumor obviously affected nomogram predictive accuracy. More important, the nomogram could be used by physicians to identify patients by calculating their probability of postoperative patients with CRC, and to offer appropriate therapy and optimal strategy. Therefore, patients with overexpression of 91H should be closely monitored and received appropriate adjuvant therapies after CRC resection.

The overexpression of 91H was involved in cancer progression [14], [23]. However, reason why 91H was dysregulated in tumor remained unknown. Copy number variation was an important form of genomic structure alteration, contributing to certain genetic and developmental diseases, including ovarian cancer and breast cancer [31], [32]. H19/IGF2 with copy number variations, located on the 11p15 imprinted region, was reported in Beckwith-Wiedemann and Silver-Russell syndromes [33], [34]. Meanwhile, 91H gene, H19 antisense RNA, was also located in the chromosome 11p15.5 [14]. Hence, it could be speculated that overexpression of 91H in CRC may be associated with CNV. In the present study, CNV of 91H was observed in two patients with high 91H expression, and copy number deletion of 91H was occurred in one patient with CRC (Figure 2). Although the result did not show statistically significant, it provided a potential new strategy in investigating 91H-mediated gene regulation on cancer. Due to the limited size of cases enrolled in this study, the further study was need in future to illustrate the potential relationship.

Some limitations of this study should be acknowledged. Firstly, the number of patients with CRC was not large. Further studies should be performed to verify the association between 91H expression and CRC, and to determine whether 91H expression was associated with CNV in patients with CRC. Secondly, the follow-up periods were not enough long (range, 2–36 months), which did not accurately predict overall survival of CRC patients. Further investigations were undertaken to validate this result by prolonged follow-up of patient cohort or another cohort of patients with CRC. Finally, all in vitro assays for biological functions were only conducted in a single cell line. Despite the limitations showed in this study, these findings supported the importance of the role of 91H in various stages of cancer progression promoting metastasis of CRC.

In summary, 91H expression was significantly up-regulated in CRC tissue samples and cell lines. The elevated expression of 91H was associated with poor prognosis and distant metastasis. Moreover, 91H CNV explained only a few percent of observed overexpression. Cumulatively, these findings indicated that 91H played a vital role in the development and progression of CRC. By understanding the accurate role of 91H in the pathogenesis of CRC, a novel and promising therapeutic strategy would be developed for further CRC treatment, based on down-regulation of these oncogenic lncRNAs.

## Supporting Information

Figure S1 **The relationship between copy number variation and 91H expression in tumor tissues and adjacent normal tissues.**
(TIF)

Figure S2 91H relative expression was quantified in four CRC cell lines and a normal human intestinal epithelial cell line by qRT-PCR (mean ± SEM; ** ***P*** **<0.01).**
(TIF)

Figure S3 HCT-116 cells were transfected with si-NC and si-91H. After 48 h, 91H expression was effectively inhibited by qRT-PCR compared with si-NC (mean ± SEM; ** ***P*** **<0.01).**
(TIF)

Figure S4 **91H promoted the proliferation of HCT-116 cells via MTT assay (mean ± SEM;** ***P*** **<0.05).**
(TIF)

Figure S5 **Apoptosis was investigated using cytometric analysis at 48 h after infection with si-91H or si-NC.**
(TIF)

Figure S6 **The scratch wound assay was assessed to cell motility. The knockdown of 91H inhibited HCT-116 cell motility.**
(TIF)

Figure S7 **91H promoted invasion and migration of HCT-116 cells based on transwell assay.**
(TIF)

Figure S8 The number of cells that invaded or migrated through the chamber was evaluated in 5 fields for each experimental group and averaged. The number of invasive or migrated cells for each experimental group was counted as the average of 5 five fields of vision under a microscope (* ***P*** **<0.05).**
(TIF)

Table S1 **Patients’ information.**
(DOCX)

Table S2 **The results of qRT-PCR for each sample.**
(XLSX)
