# Correction: Up-Regulation of 91H Promotes Tumor Metastasis and Predicts Poor Prognosis for Patients with Colorectal Cancer

**DOI:** 10.1371/journal.pone.0304249

**Published:** 2024-05-17

**Authors:** Qiwen Deng, Bangshun He, Tianyi Gao, Yuqin Pan, Huiling Sun, Yeqiong Xu, Rui Li, Houqun Ying, Feng Wang, Xian Liu, Jie Chen, Shukui Wang

In Fig 12, the images are misarranged. The si-NC and si-91H panels have a similar section in transwell invasion assays. Please see the correct Fig 12 here.

**Fig 12:**
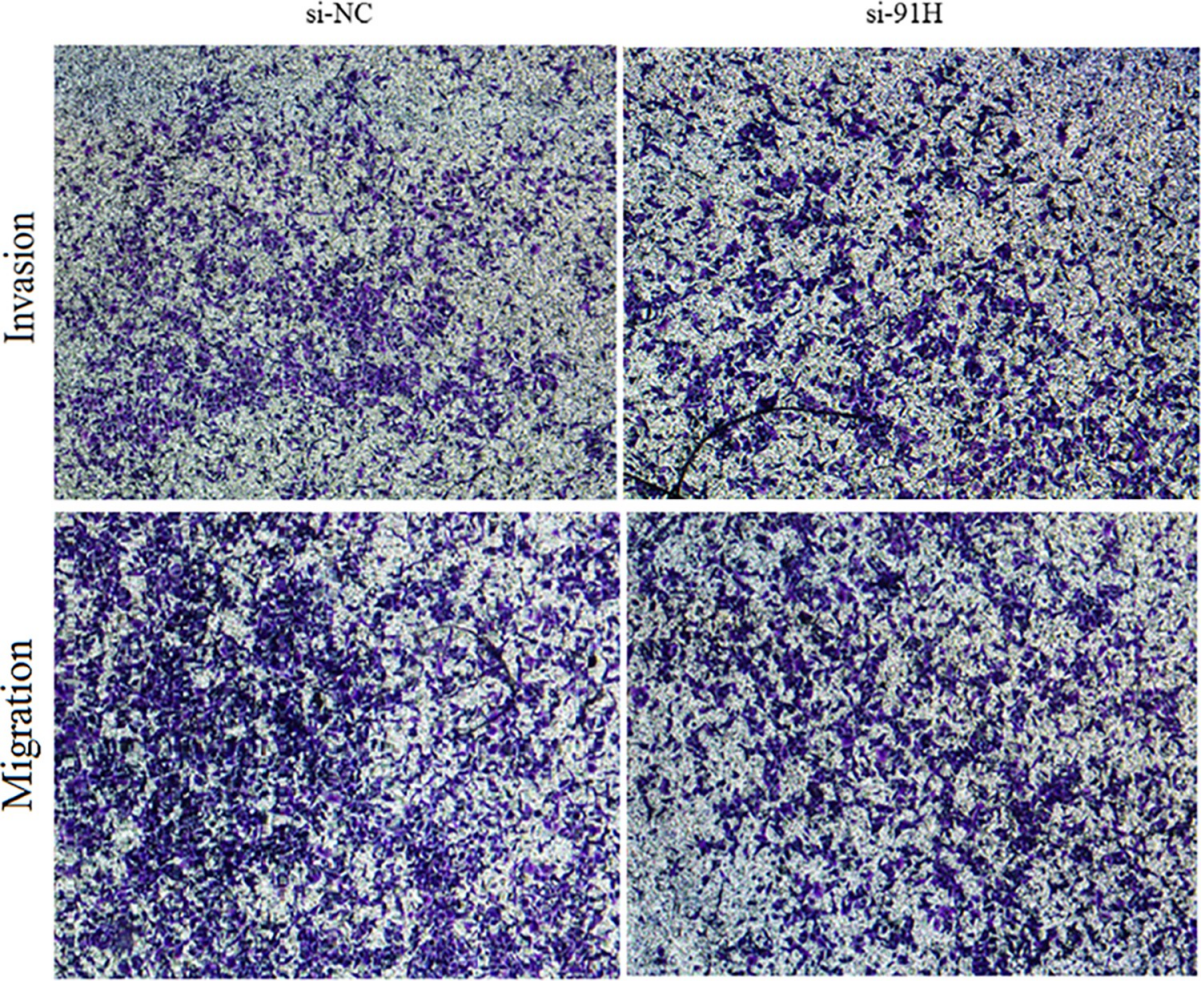
91H promoted invasion and migration of HCT-116 cells based on transwell assay.

## References

[1] Deng Q, He B, Gao T, Pan Y, Sun H, Xu Y, et al. (2014) Up-Regulation of 91H Promotes Tumor Metastasis and Predicts Poor Prognosis for Patients with Colorectal Cancer. PLOS ONE 9(7): e103022. 10.1371/journal.pone.0103022 25058480 PMC4109963

