# Supplementary figures and images for: Up-Regulation of 91H Promotes Tumor Metastasis and Predicts Poor Prognosis for Patients with Colorectal Cancer
Source: PLoS One. 2014 Jul 24;9(7):e103022. doi: 10.1371/journal.pone.0103022 (PMC4109963; doi:10.1371/journal.pone.0103022)

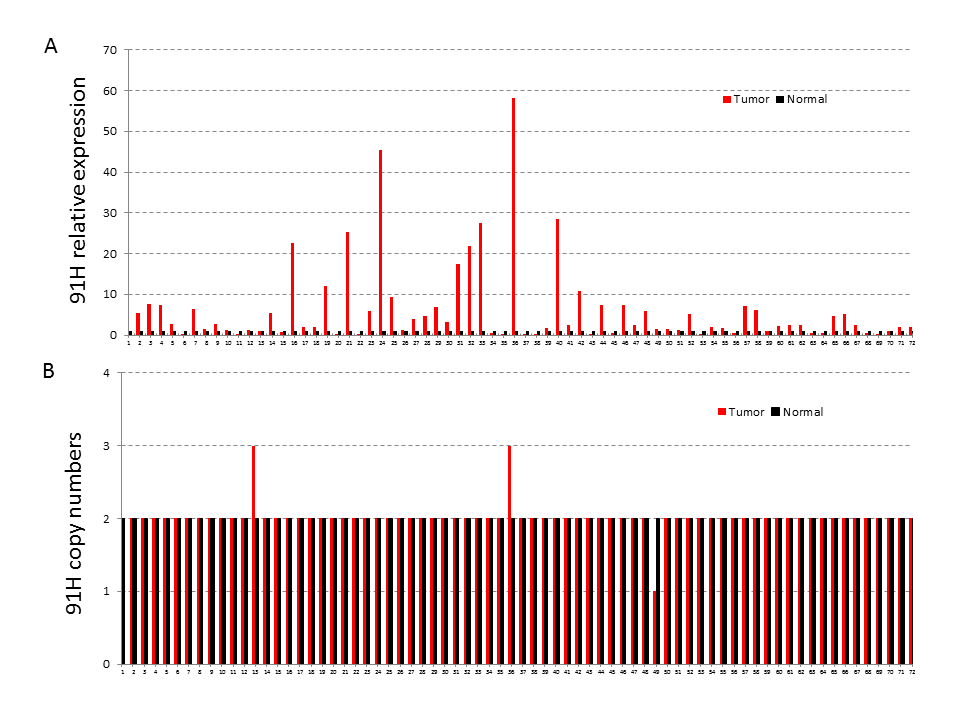

Supplement: Figure S1 — The relationship between copy number variation and 91H expression in tumor tissues and adjacent normal tissues. (TIF) [file pone.0103022.s001.tif]

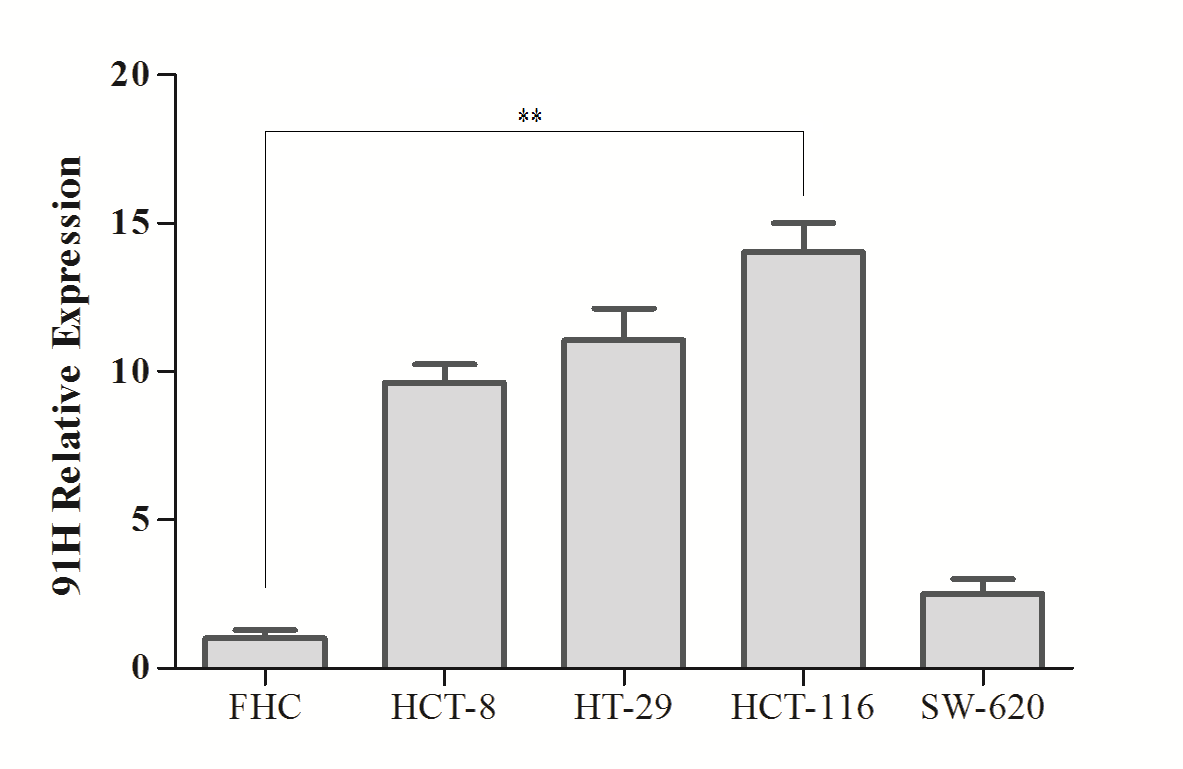

Supplement: Figure S2 — 91H relative expression was quantified in four CRC cell lines and a normal human intestinal epithelial cell line by qRT-PCR (mean ± SEM; ** P <0.01). (TIF) [file pone.0103022.s002.tif]

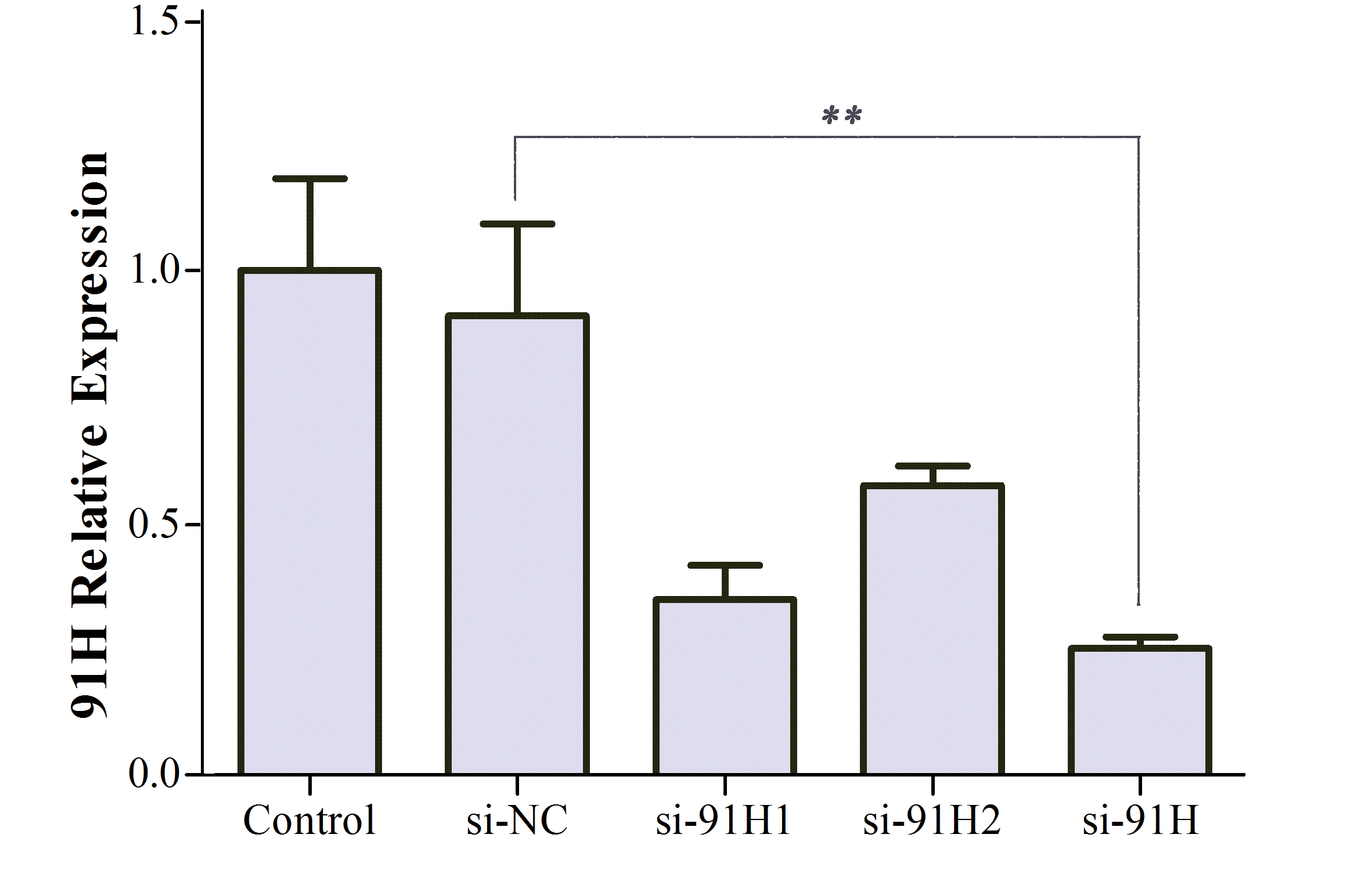

Supplement: Figure S3 — HCT-116 cells were transfected with si-NC and si-91H. After 48 h, 91H expression was effectively inhibited by qRT-PCR compared with si-NC (mean ± SEM; ** P <0.01). (TIF) [file pone.0103022.s003.tif]

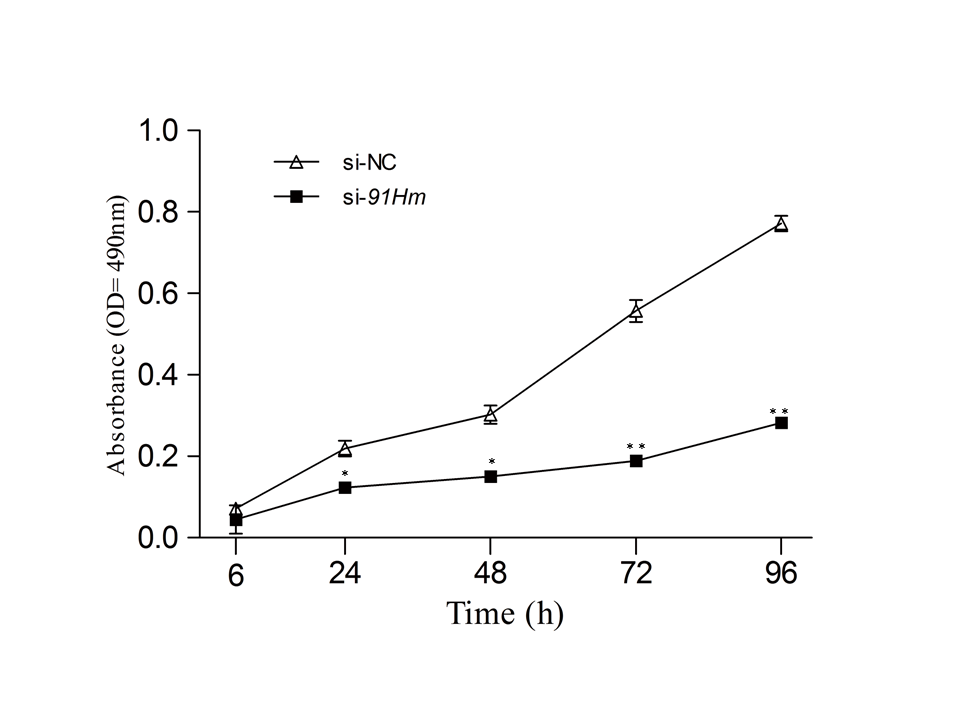

Supplement: Figure S4 — 91H promoted the proliferation of HCT-116 cells via MTT assay (mean ± SEM; P <0.05). (TIF) [file pone.0103022.s004.tif]

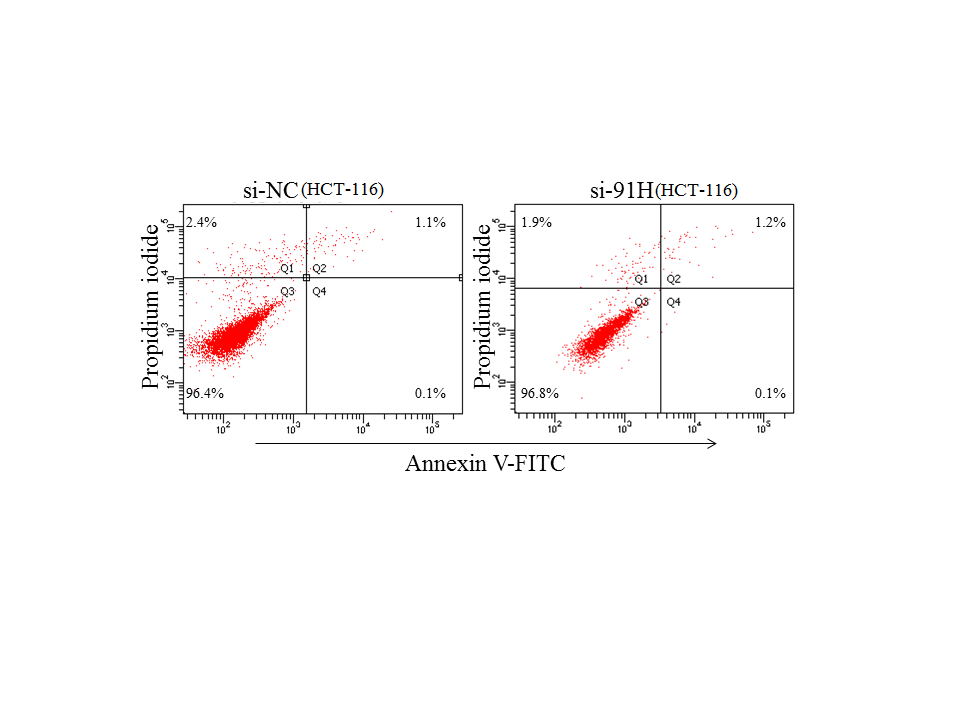

Supplement: Figure S5 — Apoptosis was investigated using cytometric analysis at 48 h after infection with si-91H or si-NC. (TIF) [file pone.0103022.s005.tif]

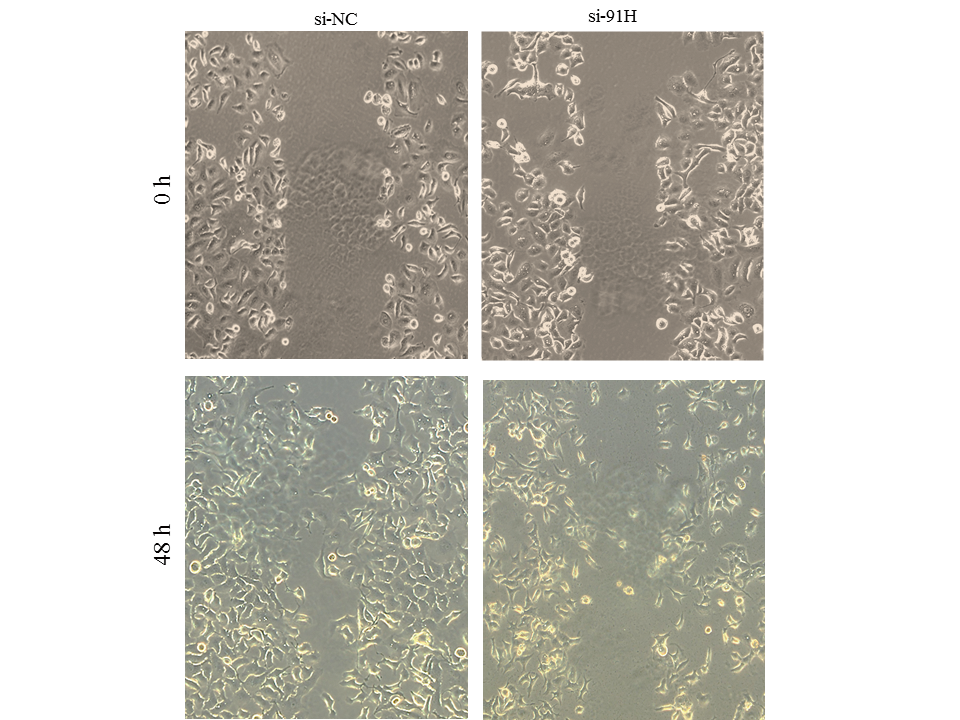

Supplement: Figure S6 — The scratch wound assay was assessed to cell motility. The knockdown of 91H inhibited HCT-116 cell motility. (TIF) [file pone.0103022.s006.tif]

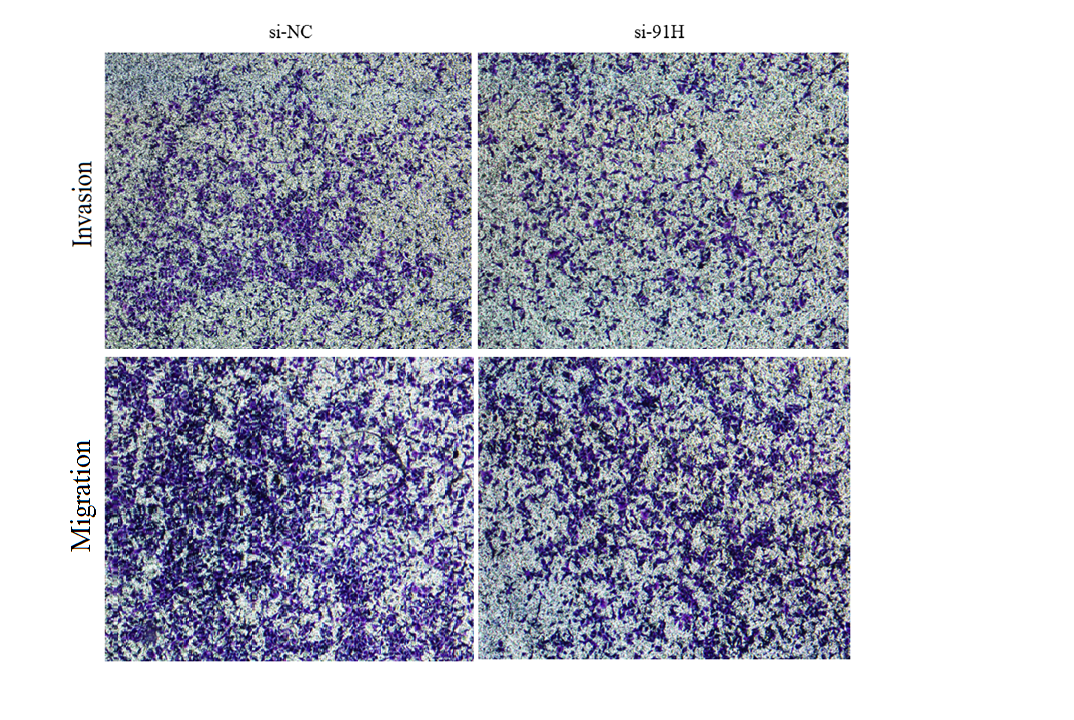

Supplement: Figure S7 — 91H promoted invasion and migration of HCT-116 cells based on transwell assay. (TIF) [file pone.0103022.s007.tif]

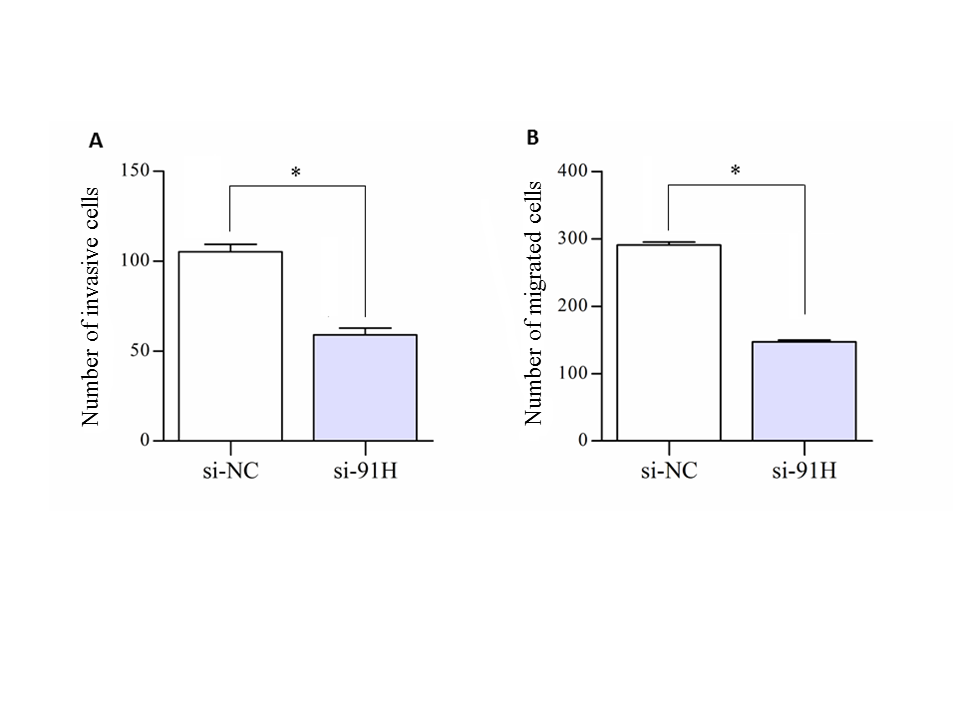

Supplement: Figure S8 — The number of cells that invaded or migrated through the chamber was evaluated in 5 fields for each experimental group and averaged. The number of invasive or migrated cells for each experimental group was counted as the average of 5 five fields of vision under a microscope (* P <0.05). (TIF) [file pone.0103022.s008.tif]
