# Supplementary material for: Up-Regulation of 91H Promotes Tumor Metastasis and Predicts Poor Prognosis for Patients with Colorectal Cancer
Source: PLoS One. 2014 Jul 24;9(7):e103022. doi: 10.1371/journal.pone.0103022 (PMC4109963; doi:10.1371/journal.pone.0103022)
Supplement: Table S1 — Patients’ information. (DOCX) [file pone.0103022.s009.docx]

Basic information of patients with CRC

| NO. | Sex | Age | Tumor location | TNM | T | N | M | Grade | time(month) | status^a^ | 91H relative expression^b^ |
| --- | --- | --- | --- | --- | --- | --- | --- | --- | --- | --- | --- |
| 1 | Female | 89 | colon | IV | 3 | 1 | 1 | G2 | 13 | 1 | 3.06 |
| 2 | Male | 79 | colon | II | 3 | 0 | 0 | G2 | 36 | 0 | 5.37 |
| 3 | Female | 70 | colon | III | 3 | 1 | 0 | G2 | 17 | 1 | 7.65 |
| 4 | Female | 76 | colon | IV | 3 | 0 | 1 | G3 | 13 | 1 | 7.39 |
| 5 | Female | 71 | colon | II | 3 | 0 | 0 | G2 | 36 | 0 | 2.66 |
| 6 | Female | 61 | rectum | III | 3 | 1 | 0 | G2 | 36 | 0 | 0.03 |
| 7 | Male | 42 | colon | IV | 4 | 1 | 1 | G2 | 8 | 1 | 6.41 |
| 8 | Male | 32 | colon | IV | 3 | 2 | 1 | G2 | 36 | 0 | 1.43 |
| 9 | Female | 62 | colon | II | 3 | 0 | 0 | G2 | 36 | 0 | 2.63 |
| 10 | Female | 85 | colon | III | 4 | 1 | 0 | G2 | 2 | 1 | 1.28 |
| 11 | Male | 74 | colon | II | 3 | 0 | 0 | G1 | 6 | 0 | 0.05 |
| 12 | Male | 78 | colon | III | 3 | 1 | 0 | G2 | 36 | 0 | 1.31 |
| 13 | Female | 65 | colon | III | 3 | 1 | 0 | G2 | 36 | 0 | 0.99 |
| 14 | Female | 42 | colon | II | 3 | 0 | 0 | G3 | 36 | 0 | 5.43 |
| 15 | Female | 58 | rectum | I | 2 | 0 | 0 | G2 | 36 | 0 | 0.70 |
| 16 | Male | 80 | colon | III | 2 | 1 | 0 | G2 | 36 | 0 | 22.63 |
| 17 | Female | 67 | colon | II | 3 | 0 | 0 | G2 | 36 | 0 | 1.91 |
| 18 | Male | 61 | colon | II | 3 | 0 | 0 | G1 | 36 | 0 | 2.06 |
| 19 | Female | 66 | rectum | II | 3 | 0 | 0 | G2 | 36 | 0 | 12.13 |
| 20 | Male | 65 | colon | I | 2 | 0 | 0 | G2 | 36 | 0 | 0.10 |
| 21 | Male | 65 | colon | II | 3 | 0 | 0 | G2 | 17 | 1 | 25.37 |
| 22 | Female | 50 | colon | III | 3 | 1 | 0 | G2 | 36 | 0 | 0.20 |
| 23 | Female | 59 | colon | III | 3 | 2 | 0 | G2 | 23 | 1 | 5.96 |
| 24 | Male | 84 | rectum | II | 3 | 0 | 0 | G1 | 36 | 0 | 45.41 |
| 25 | Male | 56 | colon | III | 3 | 1 | 0 | G2 | 36 | 0 | 9.22 |
| 26 | Female | 85 | colon | II | 3 | 0 | 0 | G2 | 36 | 0 | 1.14 |
| 27 | Male | 80 | rectum | II | 3 | 0 | 0 | G1 | 28 | 1 | 3.94 |
| 28 | Female | 82 | colon | IV | 4 | 0 | 1 | G2 | 8 | 1 | 4.77 |
| 29 | Male | 71 | colon | IV | 3 | 1 | 1 | G3 | 12 | 1 | 6.96 |
| 30 | Male | 79 | colon | IV | 4 | 0 | 1 | G3 | 18 | 1 | 3.22 |
| 31 | Male | 77 | colon | IV | 3 | 0 | 1 | G3 | 30 | 1 | 17.45 |
| 32 | Male | 83 | colon | IV | 3 | 0 | 1 | G2 | 18 | 1 | 21.86 |
| 33 | Male | 59 | colon | IV | 3 | 1 | 1 | G2 | 4 | 1 | 27.57 |
| 34 | Female | 65 | rectum | III | 3 | 1 | 0 | G2 | 36 | 0 | 0.54 |
| 35 | Male | 84 | rectum | I | 2 | 0 | 0 | G2 | 36 | 0 | 0.17 |
| 36 | Male | 68 | colon | IV | 4 | 2 | 1 | G3 | 4 | 1 | 58.28 |
| 37 | Male | 64 | rectum | II | 3 | 0 | 0 | G2 | 36 | 0 | 0.18 |
| 38 | Male | 72 | rectum | I | 2 | 0 | 0 | G1 | 36 | 0 | 0.21 |
| 39 | Male | 70 | colon | II | 3 | 0 | 0 | G2 | 36 | 0 | 1.71 |
| 40 | Male | 74 | rectum | II | 3 | 0 | 0 | G2 | 36 | 0 | 28.54 |
| 41 | Male | 79 | colon | IV | 3 | 1 | 1 | G2 | 20 | 1 | 2.52 |
| 42 | Female | 63 | colon | II | 3 | 0 | 0 | G2 | 15 | 0 | 10.89 |
| 43 | Male | 73 | colon | III | 4 | 1 | 0 | G2 | 30 | 0 | 0.18 |
| 44 | Male | 68 | colon | II | 3 | 0 | 0 | G2 | 21 | 1 | 7.49 |
| 45 | Female | 72 | colon | II | 3 | 0 | 0 | G1 | 36 | 0 | 0.53 |
| 46 | Male | 66 | rectum | III | 3 | 1 | 0 | G1 | 36 | 0 | 7.44 |
| 47 | Male | 61 | rectum | I | 2 | 0 | 0 | G3 | 36 | 0 | 2.58 |
| 48 | Female | 73 | colon | IV | 3 | 0 | 1 | G3 | 2 | 1 | 5.82 |
| 49 | Male | 85 | rectum | IV | 3 | 1 | 1 | G2 | 24 | 1 | 1.56 |
| 50 | Female | 44 | rectum | IV | 3 | 1 | 1 | G2 | 30 | 1 | 1.39 |
| 51 | Male | 46 | colon | IV | 3 | 1 | 1 | G3 | 24 | 1 | 1.35 |
| 52 | Female | 78 | colon | II | 3 | 0 | 0 | G1 | 36 | 0 | 5.21 |
| 53 | Female | 47 | rectum | III | 3 | 2 | 0 | G2 | 36 | 0 | 0.18 |
| 54 | Male | 63 | rectum | II | 3 | 0 | 0 | G2 | 36 | 1 | 1.92 |
| 55 | Male | 75 | colon | II | 3 | 0 | 0 | G2 | 36 | 0 | 1.77 |
| 56 | Male | 71 | colon | IV | 3 | 2 | 1 | G3 | 7 | 1 | 0.61 |
| 57 | Male | 74 | rectum | III | 3 | 1 | 0 | G2 | 27 | 1 | 7.01 |
| 58 | Female | 56 | colon | IV | 3 | 0 | 1 | G2 | 9 | 1 | 6.15 |
| 59 | Female | 69 | colon | II | 3 | 0 | 0 | G2 | 36 | 0 | 0.89 |
| 60 | Male | 60 | rectum | II | 3 | 0 | 0 | G1 | 8 | 1 | 2.20 |
| 61 | Male | 65 | colon | I | 2 | 0 | 0 | G2 | 36 | 0 | 2.59 |
| 62 | Female | 75 | colon | IV | 3 | 0 | 1 | G3 | 24 | 1 | 2.45 |
| 63 | Male | 42 | rectum | III | 3 | 1 | 0 | G3 | 23 | 1 | 0.48 |
| 64 | Female | 67 | colon | II | 3 | 0 | 0 | G1 | 24 | 0 | 0.42 |
| 65 | Male | 61 | colon | II | 3 | 0 | 0 | G2 | 6 | 0 | 4.74 |
| 66 | Male | 80 | colon | II | 3 | 0 | 0 | G2 | 14 | 1 | 5.19 |
| 67 | Male | 67 | colon | III | 4 | 1 | 0 | G2 | 15 | 0 | 2.45 |
| 68 | Male | 75 | colon | II | 3 | 0 | 0 | G1 | 36 | 0 | 0.43 |
| 69 | Female | 61 | rectum | I | 1 | 0 | 0 | G1 | 36 | 0 | 0.16 |
| 70 | Female | 78 | colon | II | 3 | 0 | 0 | G2 | 36 | 0 | 1.06 |
| 71 | Female | 82 | colon | III | 3 | 1 | 0 | G3 | 36 | 0 | 1.93 |
| 72 | Male | 45 | colon | III | 3 | 1 | 0 | G2 | 36 | 0 | 1.93 |

^a^1=death, 0=alive or loss of follow-up; ^b^91H relative expression=cancerous/noncancerous
